# Design and Baseline Findings of a Multi-site Non-randomized Evaluation of the Effect of a Health Programme on Microfinance Clients in India

**DOI:** 10.5539/gjhs.v6n1p43

**Published:** 2013-10-12

**Authors:** Somen Saha

**Affiliations:** 1 Indian Institute of Public Health Gandhinagar, Thaltej, Ahmedabad, Gujarat, India; 2 Nossal Institute for Global Health, University of Melbourne, Carlton, Victoria, Australia

**Keywords:** microfinance, awareness, sanitation, out-of-pocket medical expenditure, India

## Abstract

Microfinance is the provision of financial services for the poor. Health program through microfinance has the potential to address several access barriers to health. We report the design and baseline findings of a multi-site non-randomized evaluation of the effect of a health program on the members of two microfinance organizations from Karnataka and Gujarat states of India. Villages identified for roll-out of health services with microfinance were pair-matched with microfinance only villages. A quantitative survey at inception and twelve months post health intervention compare the primary outcome (incidence of childhood diarrhea), and secondary outcome (place of last delivery, toilet at home, and out-of-pocket expenditure on treatment). At baseline, the intervention and comparison communities were similar except for out-of-pocket expenditure on health. Low reported use of toilet at home indicates the areas are heading towards a sanitation crisis. This should be an area of program priority for the microfinance organizations. While respondents primarily rely on their savings for meeting treatment expenditure, borrowing from friends, relatives, and money-lenders remains other important source of meeting treatment expenditure in the community. Programs need to prioritize steps to ensure awareness about national health insurance schemes, entitlement to increase service utilization, and developing additional health financing safety nets for financing outpatient care, that are responsible for majority of health-debt. Finally we discuss implications of such programs for national policy makers.

## 1. Introduction

Poor health contributes to the persistent incidence of high poverty levels in India, with health expenditures driving 39 million families into poverty each year (Selvaraj & Karan, 2009). While the Government of India is poised to radically increase its share of the overall health spending (from 1.4 to 2.5 percent of gross domestic product) in its twelfth five-year plan (2012-17), and move towards providing universal health coverage for its citizens, addressing high out-of-pocket expenditure on treatment — which accounts for more than three-quarters of health spending in India (Balarajan, Selvaraj, & Subramanian, 2011) — remains a challenge. Women are three times more likely than men to go without treatment for long-term ailments (Iyer, Sen, & George, 2007) and among infants, those in the poorest two quintiles are twice as likely to die before their first birthday compared to infants in the richest quintile.

While women share a disproportionate burden of illness in India, many of them are small-scale entrepreneurs, and organized around microfinance. India is home to more than half of global microfinance clients (Mayes & Reed, 2012). Simply defined, microfinance is the provision of financial services such as loans, savings and insurance products to the poor, who lack access to formal banking services. Transactions are structured so that the clients, usually women, organize together, mostly as Self Help Groups (SHG), to repay loans and deposit savings. The group solidarity generated as a result of womens’ participation in microfinance generates social capital (Pronyk et al., 2008) which can be used to improve health (DeLoach & Lamanna, 2011; Nobles & Frankenberg, 2009). Thus, with outreach to more than 93 million members in India, microfinance provides a promising platform for addressing a critical development challenge, that of providing the rural poor access to health services, products and information. Such integrated packages may provide both the means and the knowledge to address priority health concerns, present the possibility of substantial cost recovery for microfinance providers, and allow broader outreach to target groups (Pronyk, Hargreaves, & Morduch, 2007).

A clustered randomized trial, among the indigenous communities of Jharkhand and Odisha states of India found that newborn babies born to mothers who are the main decision-makers within their households in SHG microfinance communities, had a significantly improved likelihood of surviving the first six weeks of their lives in response to the intervention, compared to babies born to analogous households in non-SHG communities (Montalvao et al., 2011; Tripathy et al., 2010). The Intervention with Microfinance for AIDS & Gender Equity (IMAGE) assessed a structural intervention that combined a microfinance programme with a gender and HIV training curriculum among rural women in Limpopo Province, South Africa. Effect estimates suggest that, relative to a matched comparison group, IMAGE participants experienced a 55 percent reduction in the past year experience of physical and/or sexual intimate partner violence (Pronyk et al., 2006). In Bangladesh, participation in a microcredit forum is seen to have a significant positive effect on maternal knowledge of prenatal care (Hadi, 2001) and increase in contraceptive use with decline in fertility (Amin, St Pierre, Ahmed, & Haq, 2001). It is observed that health education alone, usually delivered during the routinely scheduled microfinance group meetings, can improve knowledge leading to positive health-related behavioural change (Leatherman & Dunford, 2010). Health programmes by microfinance institutions have a positive impact on under-nutrition and diarrhoea, which are the most common causes of illness and childhood deaths in the developing world (Johnson & Rogaly, 1997; Marcus, Porter, & Harper, 1999).

While evidence has shown the positive effect of microfinance health programmes on access to improved sanitation services, availability of family planning products and services, affordable healthcare, and acceptability of services, the integration of microfinance with health programmes remains low. Despite the impressive outreach of microfinance, only 10 percent of the 93 million members in India have access to such integrated programmes (Saha, 2011). These networks of poor in the informal sector (largely women) meeting around the concept of self help and empowerment through access to credit and business education are regarded as the most exciting and least recognized resource for improving public health (Humphreys & Ribisl, 1999). Despite decades of existence and national programmes to promote SHG, strategies to link microfinance with improved health outcomes are limited to small pilot level initiatives in India. Part of the reason for this limitation has been the inadequate understanding of the impact of such programmes in improving access to health services. Leatherman et al. (2012) in reviewing evidence of integrated microfinance and health strategies conclude that the available evidence is of uneven strength. Their review identifies the weaknesses of applied research in this field. Notable gaps pertain to the study of health interventions that are currently integrated with microfinance programmes, or interventions which have the potential for integration. More scarce is the evidence of impact on primary healthcare, particularly in the Indian context. The challenges lie in analyzing the impact of such programmes in addressing health systems access barriers and deriving the lessons learned for effective scale-up. Understanding the intervention factors is equally important. We designed a multi-site non-randomized evaluation to assess the role of SHGs, organized around microfinance institutions with associated health programmes, in reducing barriers to access to health care for the poor. Baseline survey was conducted during 2012, and discussed in this paper. The follow-up survey is scheduled after one year of programme implementation, as detailed in the following section. This paper describes the study design, the methods used to evaluate it and provides baseline data from the study.

## 2. Methods and study design

### 2.1 Aims

The aim of the study is to evaluate the effect of targeted health programmes through microfinance groups in reducing barriers to access health care for the poor. We hypothesize that:

- Between baseline and the end of the intervention after 12 months, significant differences in the primary outcome - ‘incidence of childhood diarrhoea in two weeks preceding the survey’ - between the intervention and control groups will be identified;
- Between baseline and the end of the intervention after 12 months, significant differences in the secondary outcomes - ‘incidence of institutional delivery’, ‘toilet facilities at home’, and ‘out-of-pocket expenditure on medical treatment during four weeks preceding the survey’ - between the intervention and control groups will be identified.

### 2.2 Study Setting

The study has been planned as repeated data collection, at two time periods, among SHG members of Self Employed Women’s Association (SEWA) in Gujarat, and Shri Kshethra Dharmasthala Rural Development Project (SKDRDP) in Karnataka. Together, the two organizations reach out to 2.4 million poor.

### 2.3 Health Intervention

Both the organizations provide financial services to its members through savings, loans and insurance. A portion of their members receive health services pertaining to access to healthcare at doorstep, health awareness, better nutrition, improved work and living environment, and health insurance. At SEWA the integrated health services are provided by a team of dedicated volunteers (*aagewans*) and paramedical staff (barefoot doctors and mid-wives) at the doorstep of the members. Health workers are trained on delivery of cheap medicines and primary health care services. Communities are made aware of their (mainly maternal and child health) health and nutrition needs and basic hygiene by these trained health workers. The organization also offers a health insurance package to its members, including maternity benefits (Aggarwal, 2008). At SKDRDP, field animators or *sevanirathas* mobilize communities around health and sanitation, education, housing, livelihoods and microfinance. Sampoorna Suraksha, a unique comprehensive insurance programme provides financial assistance to meet the medical expenses of the member and family. In all the study areas, the organizations reach out with access to health services to some, but not all, of their SHG members. Availability of health services is based on operational and financial factors at the level of the organization rather than on the behaviour of individual clients. During early 2012, 125 new villages within the study talukas were selected for health promotion activities.

### 2.4 Inclusion Criteria

We interviewed women SHG members in the reproductive age group from the study area, having a child aged less than two years at the time of the survey.

#### 2.4.1 Recruitment, Inclusion Criteria and Community Matching

Unless great care is taken, the design of evaluation studies relating to public health interventions, such as health promotion interventions, is susceptible to engendering invalid results and, therefore, requires multiple, flexible, and community driven strategies (Rychetnik, Frommer, Hawe, & Shiell, 2002). For the purposes of this impact evaluation study, SHG members in villages with access to health services are to be compared with SHG members from villages without access to these services. Prior to the selection of individuals to participate in the study, each village with health interventions was pair-matched with a village not receiving health interventions. Pair-matching ensured that the villages were from the same administrative area, but did not share a boundary. We also used a village classification developed by the local organizations based on socio-economic indicators of the villages to match the intervention and comparison villages. In the absence of randomization, a pair matched study design addresses key threats to validity which include contamination due to mixing of groups due to proximity, and other existing health programmes that might affect the results.

A sampling frame comprising all members of the SHG in the intervention and comparison communities was generated. The inclusion criteria were women in the reproductive age group, having a child aged less than two years at the time of the survey. Consenting women meeting the inclusion criteria were selected using systematic random sampling. For every woman in an intervention village, a woman was selected from a matched village, not exposed to a health programme. The inclusion and matching criteria, and recruitment procedures to both intervention and comparison cohorts are provided in detail in Table 1.

**Table 1:** Recruitment process for the study

Study Arm	Inclusion criteria	Recruitment procedure
Intervention group	Women in reproductive age group, having a child less than two years at the time of survey, member of SHG from a village in the expansion area of the MFI health programme.	Consenting women residing in the intervention community, and from villages in the expansion phase of MFI health program
Comparison group	Women in reproductive age group, having a child less than two years at the time of survey, member of SHG from a village not exposed to a health programme	For every woman in an intervention village, a woman was selected from a matched village, not exposed to a health programme.
	Matching:	
	i. similar socio-economic status	
	i. same administrative area	
	i. intervention and comparison villages does not share a boundary.	

**Figure 1:**
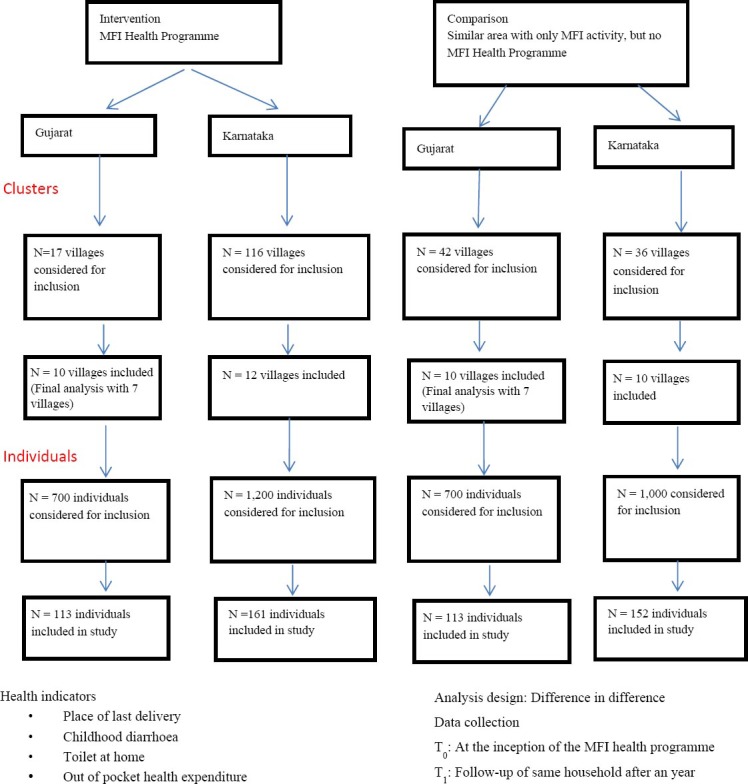
Research design and analysis plan

### 2.5 Analysis Strategy for Impact Evaluation

We will measure the difference in the two time periods, before and after the health programme, among the same households. Results from the baseline survey will be compared with a follow-up survey after one year of programme implementation among the intervention and comparison groups. We will regress the effect of microfinance and health programme in influencing outcome controlling for socio-economic status, and village fixed effect.

Additionally, in the follow up phase of the study, to assess the programme impact factors, we will conduct interviews with programme managers, and focus group discussions with representative women SHG members from the intervention villages. Participants will be asked questions on trust relations within the community, social capital, and the contribution of the health programme in addressing the primary health-related outcomes measured through this study.

### 2.6 Sample Size

The study follows the standard practice of finding the sample size that gives a 95 percent chance of rejecting the null hypothesis of zero change in outcome indicators with 80 percent power, and design effect of 1.5 for cluster survey, and assumes 20 percent non response rate. The indicator for calculation of sample size was children suffering with diarrhoea in the last two weeks, and given treatment. Least measures for Karnataka (75.8) and Gujarat (65.7), obtained from District Level Health Survey 3, were used for sample size estimation. Childhood diarrhoea measure is taken as a proxy for health status, measured through MFI linked health intervention. It is more amenable to change, and the effect can be measured through a survey questionnaire within a one-year span. Projected samples for individuals to be enrolled are given in Table 2.

**Table 2:** Sample size for baseline data

	**Intervention communities**	**Comparison communities**
Gujarat	156	156
Karnataka	156	156

Interviews with selected women who consented to participate were conducted in the local languages by women field investigators from the local communities, after receiving extensive training, and pretesting of questionnaires. Questions were asked on primary outcome measures and on additional contextual variables. Interview questions on primary outcome measures included questions on diarrhoea among children less than two years in last two weeks, medical care for diarrhoea, availability and use of toilet, place of last delivery, and questions on type of illness in last four weeks, expenditure on medical care, and coping mechanisms. In the case of questions related to knowledge of services or illness, interviewers were specifically instructed not to prompt for responses, but to encourage participants to answer from their own knowledge and beliefs.

### 2.7 Ethics

The interview process was explained to the participants in plain language. Before initiating the survey, written informed consent was obtained from eligible women willing to be part of the study. As all members in the group were literate, verbal or witnessed consent process was considered, but deemed not required. The research protocol for this study, including the process of obtaining informed consent, has received approval from the Institutional Ethics Committee of the Public Health Foundation of India, New Delhi, India (TRC-IEC-124/12, dated 13 April 2012), as well as Human Ethics Advisory Group at Nossal Institute for Global Health, University of Melbourne, Australia (Ethics Id: 1239067.1).

## 3. Results of Baseline Survey

### 3.1 Characteristics of Participants

A total of 639 members were interviewed in the study: 322 in the intervention arm (161 from Gujarat, and 161from Karnataka), and 317 in the comparison arm (165 from Gujarat, 152 from Karnataka). However, during analysis, three villages from the intervention arm and three pair matched villages from the comparison arm had to be dropped in Gujarat. Hence, the final analysis of Gujarat is reported for 113 participants each in the intervention and comparison arms.

The socio-demographic characteristics for the overall study population are listed in Table 3. Median age of respondents from Karnataka was five years more than those from Gujarat. Respondents from Gujarat were more likely to reside in *kaccha* houses (with mud walls and thatched roofs) and work in agricultural activities. Median monthly household expenditure was Rs. 4000 (73 USD).

**Table 3:** Characteristics of participants

	Karnataka		Gujarat		Chi-squared p-value
	Intervention (N=161)	Comparison (N=152)	Intervention (N=113)	Comparison (N=113)	
Age in years of SHG members (median, IQR)	30 (9)	31(9)	26 (7)	25 (7)	0.6*
Education					0.3
No formal education	28 (17.7%)	37 (24.6%)	34 (29.9%)	21 (18.7%)	
1-8 grade	62 (38.6%)	67 (44.4%)	49 (43.3%)	59 (52.4%)	
9-12 grade	59 (36.7%)	39 (25.4%)	21 (18.9%)	22 (19.3%)	
More than 12 grade	11 (7.0%)	9 (5.6%)	9 (7.9%)	11 (9.6%)	
Occupation of SHG members					0.3
Farm labor	28 (17.1%)	31 (20.4%)	44 (39.1%)	29 (25.3%)	
Construction labor	6 (3.8%)	5 (3.5%)	-	-	
Factory worker	6 (3.8%)	10 (6.3%)	-	-	
Service (govt./private)	5 (3.1%)	5 (3.5%)	5 (4.3%)	5 (4.8%)	
Self employed Business/shop	27 (16.5%)	22 (14.8%)	1 (0.6%)	1 (1.2%)	
Housewife	37 (22.8%)	32 (21.1%)	36 (32.3%)	47 (41.6%)	
Not reported	53 (32.9%)	46 (30.4%)	27 (23.7%)	30 (26.5%)	
Monthly expenditure in INR (median, IQR	4000 (2000)	4000 (2000)	4000 (2000)	4000 (2000)	0.4
Type of House					0.1
Pucca House	38 (23.6%)	36 (23.7%)	37 (32.9%)	49 (43.0%)	
Semi-pucca house	118 (73.3%)	96 (63.2%)	42 (37.3%)	36 (31.5%)	
Kaccha House	5 (3.1%)	20 (13.2%)	34 (29.8%)	29 (25.5%)	
Source of water					0.9
Private tap	56 (35.0%)	56 (36.9%)	109 (96.9%)	106 (93.9%)	
Public tap	40 (24.9%)	41 (26.8%)	4 (3.1%)	5 (4.3%)	
Private hand pump	3 (1.9%)	1 (0.7%)	-	1 (0.6%)	
Public hand pump	3 (1.9%)	1 (0.7%)	-	-	
Tube well	7 (4.4%)	4 (2.7%)	-	1 (0.6%)	
Well/river/pond	51 (31.9%)	49 (32.2%)	-	1 (0.6%)	

* Wilcoxon equality of medians test between intervention and comparison group.

### 3.2 Baseline Measures of Study Outcome Variables

Except for money spent on treatment, there were no significant differences between groups with respect to age, occupation, place of last delivery, and childhood diarrhea — suggesting that the matching at baseline between intervention and comparison groups was successful (Table 4). Chi square value is pooled estimate for intervention and control site. Wilcoxon equality of medians test between intervention and comparison group are reported for certain variables where median score is reported.

**Table 4:** Baseline measures of study outcome variables

	Karnataka		Gujarat		Chi-squared p-value
	Intervention (N=161)	Comparison (N=152)	Intervention (N=113)	Comparison (N=113)	
Treat water for drinking	121 (89.8%)	123 (88.7%)	112 (99.4%)	111 (98.2%)	0.5
Toilet at home	129 (81.9%)	84 (58.9%)	44 (39.0%)	58 (51.2%)	0.1
Been sick in last 4 weeks	55 (34.8%)	59 (43.8%)	93 (82.5%)	83 (74.2%)	0.6
Sought care for sickness	53 (33.5%)	58 (41.7%)	64 (57.8%)	62 (54.5%)	0.4
Admitted in last 6 months	34 (21.3%)	43 (29.1%)	27 (22.4%)	24 (19.5%)	0.5
Money spent on treatment in last 6 months in INR (median, IQR)	2000(1800)	3650 (2300)	3000 (2200)	3000 (2200)	0.0*
Source of meeting treatment expenditure					
Savings	55 (36.0%)	58 (42.0%)	63 (59.0%)	69 (41.0%)	
Borrow from friends	20 (12.0%)	5 (4.0%)	18 (18.0%)	17 (14.0%)	
Borrow from relatives	13 (8.0%)	17 (13.0%)	29 (25.0%)	20 (20.0%)	
Health insurance	17 (11.0%)	7 (5.0%)	2 (3.0%)	3 (4.0%)	
Borrow from money lenders	6 (4.0%)	14 (9.0%)	14 (11.0%)	8 (7.0%)	
Borrow from group savings	8 (5.0%)	6 (4.0%)	2 (3.0%)	7 (4.0%)	
Business loan from MFI	-	4 (3.0%)	2 (3.0%)	1 (1.0%)	
Selling assets	2 (1.0%)	2 (1.0%)	2 (2.0%)	1 (1.0%)	
Place of last delivery					0.5
Home	28 (22.5%)	29 (23.7%)	19 (16.8%)	14 (12.1%)	
Government hospital/health centre	42 (33.3%)	49 (39.7%)	55 (49.1%)	57 (50.3%)	
Private nursing home/hospital	56 (44.2%)	45 (36.6%)	39 (34.2%)	42 (37.6%)	
Initiation of breastfeeding					0.2
30 minutes	79 (61.4%)	57 (47.3%)	58 (68.6%)	57 (68.9%)	
1 hour	19 (15.2%)	29 (24.0%)	18 (20.7%)	21 (25.2%)	
2 hours	30 (23.4%)	34 (28.7%)	9 (10.7%)	5 (5.9%)	
Colostrum fed to baby	70 (61.2%)	58 (60.6%)	56 (50.9%)	64 (55.5%)	0.3
Child suffered from diarrhea in last 2 weeks	33 (24.4%)	27 (21.2%)	41 (39.7%)	38 (34.2%)	0.3
Child needed hospitalization for diarrhea	26 (19.8%)	22 (19.5%)	4 (6.3%)	8 (8.8%)	0.8

* Wilcoxon equality of medians test between intervention and comparison group.

While treating water for drinking is nearly universal, respondents from Gujarat reported low use of toilets at home (39.0% in intervention arm, and 51.2% in comparison arm). About half of the respondents from Gujarat reported having been sick in the four weeks preceding the survey (57.8% in intervention arm, and 54.5% in comparison arm), compared to about a third in Karnataka. Malaria/fever was reported as the major illness, followed by respiratory illnesses. Money spent on treatment ranged between INR. 2,000 - 3,650 (40-73 USD) per month. While respondents primarily relied on their savings for meeting treatment expenditure, more than a third had to borrow money from friends, relatives or money lenders to meet treatment cost. A fifth of all deliveries continued to be conducted at home. While the majority of the respondents reported early initiation of breastfeeding, half of respondents from Gujarat, and third from Karnataka did not feed colostrum to the newborns.

No statistically significant differences were found between the intervention and the control group for the primary outcome variable: incidence of childhood diarrhea (p=0.26), and the secondary outcome variables: institutional delivery (p=0.49), and toilet at home (p=0.08), with the exception of the variable of out-of-pocket expenditure on medical treatment, which was significantly more in the control group (median 2,800 in intervention group vs. 3,200 in control group, p<0.05).

## 4. Discussion

We have described the design of a non-randomized study to estimate the impact of health-interventions. The main strength of the study lies in its design. We used an impact evaluation design that follows a natural experiment in a real world setting, while maintaining independence from the program implementation. Pair-matching the intervention and comparison cluster ensures a close approximation in absence of a classical randomized control trial design. The design is easily replicable, and will provide empirical evidence about the effectiveness of this model of care. This design has certain threats to validity which includes contamination due to mixing of groups, and other existing health programs that might affect the results. To address this, comparison groups were identified to ensure geographical separation (no nearby villages), and matching villages on the basis of socio-economic indicators. Since both the comparison and intervention groups were from the same state, any effect due to national/state health programs was adjusted. The design imbalance in money spent on treatment is primarily in Karnataka, and we will adjust this in the final difference-in-difference analysis through adjusting for socio-economic factors.

We also present the findings of the baseline portion of our survey. Our findings show the intervention and comparison groups are similar on key study variables. This is an important criterion to attribute the effects of treatment on outcome variable (Maxwell, 1973; Meinert, 2012).

Low reported use of toilet at home is an area that would require urgent program attention. Efforts made at meeting the sanitation challenges have been found to have very limited results. According to the latest census data, nearly half of India’s 1.2 billion people have no toilet at home, but more than half of India's people own a mobile phone (Ahmed, 2012). Field studies indicate that even the use of the existing toilets in both rural and urban areas is very low. WHO/UNICEF’s Joint Monitoring Program on sanitation for the Millennium Development Goals indicated that 59% (626 million) Indians still do not have access to toilets and practice open defecation and that majority of them live in rural areas (WHO, 2012). This should be an area of program priority, solutions to which can include intensive behavior change communication, connecting the community with government assistance schemes for toilet construction, as well as soft loans for building toilets at home. Besides the loan eligibility criteria of the microfinance program can promote practice of open-defecation free households, as was adopted by BRAC (Hadi, 2001). While respondents primarily rely on their savings for meeting treatment expenditure, borrowing from friends, relatives, and money-lenders remains other important source of meeting treatment expenditure in the community. Most of India’s estimated 1.2 billion people have to pay for medical treatment out of their own pockets, resulting in health-care debt. As a response several community health insurance schemes promoted by nongovernment organizations (including the two organizations under study) were promoted as a transitory measure towards universal health care, and to provide protection against out-of-pocket expenditure for households. Of recent the Planning Commission of Government of India envisaged steps towards achieving universal health care. However, the biggest challenge is getting this unregulated and unorganized system under a single umbrella for providing universal access (Durairaj, 2010). Along with bringing the poor in the safety net of health insurance, organizations can take additional steps to ensure awareness of entitlement to increase service utilization, and developing additional health financing safety nets like loans or savings for financing most of outpatient care, that are responsible for majority of health-debt.

Finally, this integrated delivery model presents a development alternative in addressing multiple barriers to health access for the poor, organized around microfinance, self help groups and other livelihood promotion programs. These groups and their federated structure can complement existing public health program, create demand for services in the community, and perform community-based monitoring of health services. Our research is designed not only to provide evidence of effectiveness of this model of care, but also an understanding of the intervention factors that contribute to the outcome.
